# Factors associated with child survival in children admitted to outpatient therapeutic program at public health institutions in Afar Regional State, Ethiopia: a prospective cohort study

**DOI:** 10.1186/s41043-019-0193-1

**Published:** 2019-11-27

**Authors:** Misgan Legesse Liben, Abel Gebre Wuneh, Reda Shamie, Kiros G/her

**Affiliations:** 1 Department of Public Health, College Health Sciences, Woldia University, Woldia, Amhara Ethiopia; 2 Department of Public Health, College of Medical and Health Sciences, Samara University, Semera, Afar Ethiopia; 3 School of Public Health, College of Health Sciences, Mekelle University, Mekelle, Ethiopia

**Keywords:** Outpatient therapeutic, Prospective, Cohort, Afar, Ethiopia

## Abstract

**Introduction:**

About 20 million children suffer from severe acute malnutrition each year. The World Health Organization recommends the outpatient therapeutic program as a standard treatment protocol for the management of uncomplicated severe acute malnutrition and for children who are transferred from inpatient cares after recovery. This study aimed to assess the treatment outcome of severe acute malnutrition and determinants of survival in children admitted to outpatient therapeutic program at public health institutions, Afar Regional State.

**Methods:**

Institution-based prospective cohort study was conducted on 286 children aged 6–59 months admitted to the outpatient therapeutic program, from April to September 2017, at selected public health institutions in Afar Regional State. For the comparison of time to recovery among the different groups of children on the outpatient therapeutic program, Kaplan-Meir curve was used and significance test for these differences was assessed by the log-rank test. Then, a proportional hazard in the Cox model was used to identify independent predictors of survival. *p* value < 0.05 was considered significant.

**Results:**

Of 286 children, 238 (83.2%; 95% CI (79, 88)), 18 (6.3%), 14 (4.9%), 8 (2.8%), and 8 (2.8%) cases were cured, defaulters, non-responder, died, and transfer to inpatient care, respectively. The overall mean rate of weight gain was 10.5(± 3.45) g/kg/day, and mean length of stay was 44.15(± 8.77) days. The recovery rate of children whose mothers travel less than 2 h to the health institution was about three times (AHR, 2.91; 95% CI (2.18, 3.88)) higher than children whose mothers travel 2 h and above. Compared with children who received vitamin A supplementation, children who lack supplementation were less likely (AHR, 0.39; 95% CI (0.25, 0.59)) to be cured. Moreover, the rate of recovery from outpatient therapeutic program among children who received antibiotics was about 1.4 times (AHR, 1.38; 95% CI (1.01, 1.89)) higher compared with children who did not receive of antibiotics.

**Conclusion:**

This study showed that nearly eight children in every ten had recovered from severe acute malnutrition. Therefore, considering the distance of health facility from children’s residence, improving vitamin A supplementation and antibiotics are vital in improving the rate of recovery. Further research is also required to identify and address barriers to the provision of antibiotics and vitamin A supplementation.

## Introduction

Malnutrition is a major public health problem in children aged less than 5 years. Severe acute malnutrition (SAM) is among many forms of malnutrition, specifically under-nutrition, which is defined as extremely low weight for height, by visible severe wasting (marasmus), and/or by the presence of nutritional edema (kwashiorkor). It is predominantly measured by one or more of the following criteria: weight-for-height (WFH) less than − 3 *Z*-scores; weight-for-height less than 70% of the median; mid-upper arm circumference (MUAC) less than 110 mm and presence of bilateral pitting edema [1, 2].

In 2018, over 49 million children under 5 years were wasted and nearly 17 million were severely wasted [3]. Globally, 20 million children suffer from SAM each year [4]. According to the 2008 lancet series, 10% (55 million children) of children younger than 5 years in low and middle-income countries are wasted [5]. Malnutrition is directly or indirectly associated with more than 50% of all child mortality. A child with SAM has nine times higher risk of mortality as compared with an optimally nourished child [6]. This allows SAM to be one of the top three nutrition-related causes of child mortality [7, 8].

In Ethiopia, 10% of children younger than 5 years are wasted and about 53% of all under-five deaths are attributed to malnutrition [9]*.* Over 44% of the pastoral communities in Ethiopia are food insecure where wasting was observed in the range of 8.0–27.6%, in which the highest was in Afar Region [10]. The recent demographic and health survey showed 18% prevalence of wasting in Afar Region [9]*.* In addition, wasting was 12.8% [11] and 13.8% [12] in Aysaita and Afambo districts of Afar National Regional State of Ethiopia, respectively. Hence, wasting in Afar Regional State is far from Ethiopia’s Health Sector Transformation Plan of 4.9% in children aged less than 5 years by the end of 2020 [13]. This shows that children from pastoral communities have the highest degree of nutritional vulnerability [14].

Sustainable nutrition interventions enable the fruition of a healthy and productive labor force which is vital in ensuring social and economic development [15]. The World Health Organization (WHO) recommends outpatient therapeutic program (OTP) as a standard treatment protocol for the management of uncomplicated SAM and for children who are transferred from inpatient cares after recovery [3, 16]. Currently, OTP is provided in hospitals, health posts, and health centers. The service is provided daily for new cases, and one OTP day in a week is scheduled for follow-up of enrolled cases. Children with SAM who are eligible for OTP undergo an appetite test and receive ready-to-use therapeutic foods (RUTFs) and routine medicines for administration at home. Then, they return weekly to the outpatient care site until they are discharged [17, 18].

Ready-to-use therapeutic food (RUTF) is high in energy, fortified, ready-to-eat food suitable for the treatment of children with SAM. It can be consumed easily by children from the age of 6 months without adding water. RUTF is not water-based, meaning that bacteria cannot grow in them; therefore, this food can be used safely at home without refrigeration and even in areas where hygiene conditions are not optimal [3].

In 2007 the Ethiopian Federal Ministry of Health (FMOH) in collaboration with UNICEF and WHO has developed the National Guideline for the management of SAM [17]. However, limited prospective cohort studies have been conducted on the treatment outcomes of SAM at OTP sites in Ethiopia. Furthermore, the treatment outcomes of SAM and associated factors are not well addressed in Afar Regional State. Therefore, this study aimed to assess the treatment outcome of SAM and determinants of survival in children admitted to OTP at public health institutions in Afar National Regional State, Ethiopia.

## Methods

### Study area and design

Afar National Regional State (ANRS) is one of the nine regions in the Federal Democratic Republic Ethiopia. The region is located in Northeastern part of Ethiopia bordering with four national regional states: in the north and northwest, Tigray region; in the west and south-west, Amhara region; in the south, Oromia region; and in the south-west, Somalia region. ANRS also shares international borders with Djibouti and Eritrea to the west and north-west, respectively. Administratively, the region is divided into five zones, which are further subdivided into 32 districts and 404 kebeles. The Region experienced the highest percentage (26.1%) of food-insecure households. In addition, in this region, about 41% of households consumed three or fewer food groups [19].

Institution-based prospective cohort study was conducted at public health institutions in Afar National Regional State from April to September 2017. There are 6 public hospitals, 62 health centers, and 314 health posts.

### Sample size determination and sampling procedure

A sample size of 286 was calculated using Open Source Epidemiologic Statistics for Public Health (Open Epi), Version 2.3, considering the following assumptions: 80.9 and 65.6% recovery rate among children who travel ≤ 2 h and > 2 h to the health institution, respectively [20]. Two-sided significance level (1-alpha) = 95%, power (1-beta, % chance of detecting) = 80% and ratio of sample size, unexposed/exposed = 1.

First, of the six public hospitals in Afar Regional State, three hospitals (Dubti, Kelewan, and Abala) were randomly selected by lottery method. Then, Dubti hospital refers OTP cases to Dubti health center, and Abala hospital did not have enough case flows. Therefore, Dubti hospital was replaced by Dubti health center and Abala hospital by Abala health center. All children aged 6–59 months with severe acute malnutrition who were admitted to an outpatient therapeutic program (OTP) at selected public health institutions during the study period were included. Finally, every other child was selected from the hospital and health centers.

### Data collection process and instrument

Data were collected using structured questionnaire and data extraction form. The questionnaire was prepared first in English then translated to Amharic, and back to English to check for consistency. Three health professionals currently working in each OTP sites (one in each health institution) were recruited as data collectors. The data collectors were trained for 2 days on the study instrument, consent form, how to interview, and data collection procedure. Then, the questionnaire was pretested on 5% of the sample size at Berhale health center. The pretest was done to ensure clarity, wordings, logical sequence and skip patterns of the questions. Then, amendments were made on the logical sequence of the questions. Finally, the Amharic version of the questionnaire was used to collect the data.

Mothers/caregivers of the selected children were interviewed through face to face at the health institution. Anthropometric measurements and physical examination were made. Admission and follow-up weights and MUAC were taken. Weight was measured in kilogram using hanging sprint scale and digital weight scale which were checked against a standard weight for its accuracy in a daily basis. Calibration of an instrument against zero reading was made following weighting of every child. Children were weighted with light clothing and without shoes.

Each participant on OTP had visited to the closest site weekly to receive food and a medical assessment. During every visit, the child was examined and given a weekly supply of RUTF. At each follow-up visit weight of the child, existence/extent of pitting edema, presence of disease, drugs prescribed, and outcome (death, cured, default, non-response, or transfer) were recorded on patients’ treatment cards and in the program’s register.

Finally, data were extracted using data extraction form from the cards and registration forms on every visit. The maximum follow-up period for the children on OTP was 8 weeks as recommended by Ethiopian Federal Ministry of Health (FMOH) [17]. A home visit was conducted for all children who did not return for follow-up in order to know their treatment response status.

### Study variables

The dependent variable was recovery from severe acute malnutrition (SAM). The independent variables were as follows: child characteristics (sex, age, weight, MUAC, type of admission, diarrhea, and cough at admission), household characteristics (household head, number of under-five children, family size, husband educational status), maternal characteristics (age, marital status, ethnicity, religion, educational status, occupation, parity), hospital-related characteristics (distance of health facility from child’s residence, antibiotics, vitamin A supplementation, measles vaccination, deworming, folic acid).

### Operational definition

- Cured: children who had reached the discharge criteria.
- Censored: children defaulted, non-response, died, and transferred to inpatient care were considered censored.
- Death: children who had died while she/he was in the Programme at the health facility or in transit to another component of the Programme. For the out-patient Programme, death has to be confirmed by a home visit.
- Defaulter: children who were absent for 2 consecutive weighings (14 days), confirmed by a home visit.
- Discharge criteria: W/L ≥ 85% or W/H ≥ 85% on more than one occasion (2 weeks) and no edema for 14 days.
- Non-responder: children who had not reached the discharge criteria after 2 months in the out-patient Programme.

### Data processing and analysis

Data were checked for completeness and inconsistencies. Data were cleaned, coded, and entered on to EpiData version 3.02, then exported to SPSS version 20 for analysis. Descriptive statistics were calculated for all continuous variables while frequency distribution was used to describe categorical variables.

The outcome variable was recovery/cure from sever acute malnutrition (SAM). Children defaulted, non-response, died, and transferred to inpatient care at the end of the study period were considered “censored.” Then, children who “recovered” were coded as “1” and those who were categorized as “censored" were coded as “0” for Cox regression analysis.

For the comparison of time to recovery among the different groups of children on OTP, Kaplan-Meir (KM) curve was used and significance test for these differences was assessed by log-rank test. Finally, significant variables at *p* value < 0.25 were included in multiple variable analysis of proportional hazards Cox model. The assumption for proportional hazard was assessed graphically by log-minus-log survival curve. Variables with *p* value < 0.05 in the final model were considered significant.

## Results

### Socio-demographic characteristics of study participants

Majority (71.3%) of the study participants were Afar. The mean (±SD) age of mothers/caregivers was 28.65(± 6.11) years which range from 19 to 43. Moreover, 234 (81.8%) mothers were in the age group of 20–34 years. Only 49 (17.1%) of mothers were household heads. There were 156 (54.5%) female and 130 (45.5%) male children. The mean (±SD) age of mothers/caregivers was 13.96(± 8.40) ranged from 71 to 57 months (Table 1).

**Table 1:** Socio-demographic characteristics of mothers/caregivers of children in the outpatient therapeutic feeding program in in Afar Regional State, Ethiopia, 2017

Variable	Cured *n* (%)	Censored *n* (%)
Distance of HI from residence		
< 2 h	141 (97.9)	3 (2.1)
≥ 2 h	97 (68.3)	45 (31.7)
Age of the mothers/caregivers (years)		
< 20	12 (100.0)	0 (0)
20–34	186 (79.5)	48 (20.5)
> 34	40 (100.0)	0 (0)
Maternal education status		
Non formal	231 (83.7)	45 (16.3)
Formal	7 (70.0)	3 (30.0)
Religion		
Muslim	206 (82.7)	43 (17.3)
Orthodox	32 (86.5)	5 (13.5)
Ethnicity		
Afar	164 (80.4)	40 (19.6)
Amhara	44 (93.6)	3 (6.4)
Tigray	30 (85.7)	5 (14.3)
Paternal education status		
Non formal	199 (80.6)	48 (19.4)
Formal	29 (100.0)	0 (0.0)
Household Head		
Child’s mother	48 (98.0)	1 (2.0)
Child’s father	190 (80.2)	47 (19.8)
Maternal occupation		
Housewife	217 (82.8)	45 (17.2)
Other	21 (87.5)	3 (12.5)
Marital status		
Currently married	203 (80.9)	48 (19.1)
Currently unmarried	35 (100.0)	0 (0.0)
Family size		
2	9 (100.0)	0 (0.0)
3–4	104 (87.4)	15 (12.6)
≥ 5	125 (79.1)	33 (20.9)
Parity		
1–3	135 (80.7)	32 (19.3)
4–5	62 (88.6)	8 (11.4)
≥ 6	42 (84.0)	8 (16.0)
Number of < 5 children		
1	149 (86.1)	24 (13.9)
2–3	89 (78.8)	24 (21.2)
Child’s age		
6–11	107 (87.7)	15 (12.3)
12–23	104 (79.4)	27 (20.6)
24–59	27 (81.8)	6 (18.2)
Sex		
Female	122 (78.2)	34 (21.8)
Male	116 (89.2)	14 (10.8)

### Treatment outcomes

Majority (81.5%) of children were identified as newly admitted children, and 18.5% were transferred from other health facilities (Table 2). The mean (±SD) weight of the study children at admission was 6.57(±1.69) kg while at discharge was 8.06(±1.91) kg, respectively.

**Table 2:** The distribution of comorbidities and treatments given to under-five children at OTP in Afar Regional State, Ethiopia, 2017

Variable	Cured *n* (%)	Censored *n* (%)
Type of admission		
New	208 (89.3)	25 (10.7)
Transfer in	30 (56.6)	23 (43.4)
Deworming given		
Yes	38 (88.4)	5 (11.6)
No	200 (82.3)	43 (17.7)
Measles vaccination		
No	58 (80.6)	14 (19.4)
Yes	180 (84.1)	34 (15.9)
Vitamin A supplementation		
Yes	87 (95.6)	4 (4.4)
No	151 (77.4)	44 (22.6)
Folic acid given		
Yes	5 (100.0)	0 (0.0)
No	233 (82.9)	48 (17.1)
Diarrhea at admission		
Yes	3 (33.3)	6 (66.7)
No	235 (84.8)	42 (15.2)
Cough at admission		
Yes	4 (36.4)	7 (63.6)
No	234 (85.1)	41 (14.9)
Antibiotic given		
Yes	136 (86.1)	22 (13.9)
No	102 (79.7)	26 (20.3)
Bottle feeding in 2 weeks prior to admission		
Yes	37 (60.7)	24 (39.3)
No	201 (89.3)	24 (10.7)
Colostrum feeding		
Yes	151 (83.9)	29 (16.1)
No	87 (82.1)	19 (17.9)
Prelacteal feeding		
Yes	48 (76.2)	15 (23.8)
No	129 (85.4)	22 (14.6)

Of 286 children, 238 (83.2%; 95% CI (79, 88)) were cured from severe acute malnutrition. In addition, 18 (6.3%), 14 (4.9%), 8 (2.8%), and 8 (2.8%) cases were defaulters, non-responder, died and transfer to inpatient care, respectively. The median time to recovery was 49 (19, 57) days and the mean rate of weight gain of cured children was 11.04(± 2.85) g/kg/day. The overall mean rate of weight gain was 10.5(± 3.45) g/kg/day, and mean length of stay 44.15(± 8.77) days. Mean rate of weight gain of died, defaulted, non-responder, and children transferred to inpatient care was 10.95(± 2.73), 10.56(± 5.0), 5.79(±1.65), and 1.98(± 0.55) g/kg/day, respectively. The mean length of stay in the treatment for cured, died, and defaulters was 44.71(± 7.97), 43.63(± 8.73), and 42.78(± 12.01) days, respectively. Mid upper arm circumference (MUAC) while cured, died, and defaulted was 11.48(± 0.49), 11.45(± 0.31), and 11.54(± 0.62) cm, respectively.

### Comorbidities and provision of medications

Over all 9 (3.1%) of children had diarrhea at admission. Out of 286 children, 31.8% received vitamin A supplementation, 44.8% received amoxicillin, and 1.7% received folic acid (Table 2).

### Factors associated with survival time of children on OTP

The significance of observed differences by the Kaplan-Meier survival curves among different groups of children was assessed using log-rank test. Hence, the distance of the health institution from residence, deworming, and vitamin A supplementation were statistically significant. However, the number of under-five children in the household, cough at admission, measles vaccination, and giving antibiotic were significant at *p* < 0.3. Therefore, these variables were included in multiple variable analysis of proportional hazards Cox model (Table 3).

**Table 3:** Proportional hazards Cox model multiple variable analyses of determinants of survival of children admitted to OTP in Afar Regional State, Ethiopia, 2017

Variable	Cured *n* (%)	Censored *n* (%)	AHR (95% CI)
Distance of HI from residence			
< 2 h	141 (97.9)	3 (2.1)	2.91 (2.18, 3.88)*
≥ 2 h	97 (68.3)	45 (31.7)	1
Number of < 5 children in the household			
1	149 (86.1)	24 (13.9)	0.95 (0.70, 1.27)
2–3	89 (78.8)	24 (21.2)	1
Deworming given			
Yes	38 (88.4)	5 (11.6)	0.93 (0.58, 1.49)
No	200 (82.3)	43 (17.7)	1
Vitamin A supplementation			
No	151 (77.4)	44 (22.6)	0.39 (0.25, 0.59)*
Yes	87 (95.6)	4 (4.4)	1
Cough at admission			
Yes	4 (36.4)	7 (63.6)	0.62 (0.21, 1.81)
No	234 (85.1)	41 (14.9)	1
Antibiotic given			
No	136 (86.1)	22 (13.9)	1
Yes	102 (79.7)	26 (20.3)	1.38 (1.01, 1.89)*
Measles vaccination			
No	58 (80.6)	14 (19.4)	1
Yes	180 (84.1)	34 (15.9)	1.49 (0.90, 2.46)

*Significant at *p* < 0.05

The recovery rate of children whose mothers travel less than 2 h to the health institution was about three times (AHR, 2.91; 95% CI (2.18, 3.88)) higher than children whose mothers travel 2 h and above. Compared with children who received vitamin A supplementation, children who did not receive supplementation were less likely (AHR, 0.39; 95% CI (0.25, 0.59)) to be cured. Moreover, the rate of recovery from OTP among children who received antibiotics was about 1.4 times (AHR, 1.38; 95% CI (1.01, 1.89)) higher compared with children who did not receive antibiotics.

### Comparison of time to recovery among the different groups (the KM survival curve)

**Fig. 1:**
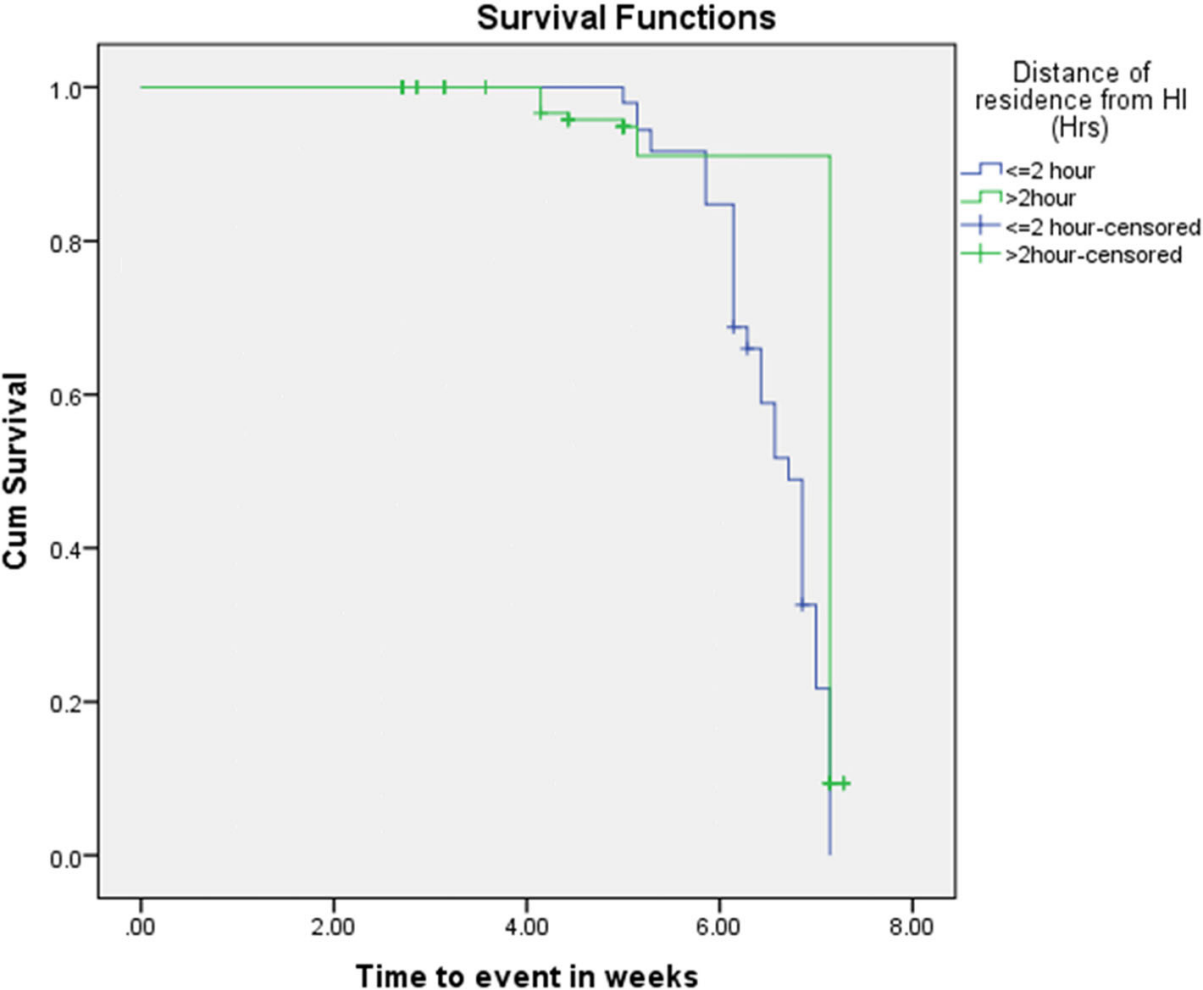
Kaplan-Meier survival curves by distance of their residence from health institution in Afar Regional State, Ethiopia, 2017

The Kaplan-Meir (KM) survival curve of distance of health institution from the residence of study children in relation to time to event shows that those who travel for less or equal to 2 h had better treatment outcomes of OTP (cure rate of 95.6%, median length of stay of 46 days and mean weight gain of 10.7 g/kg/day) compared with those who travel more than 2 h (cure rate of 77.4%, median length of stay of 49 days, and mean weight gain of 10.4 g/kg/day) (Fig. 1).

**Fig. 2:**
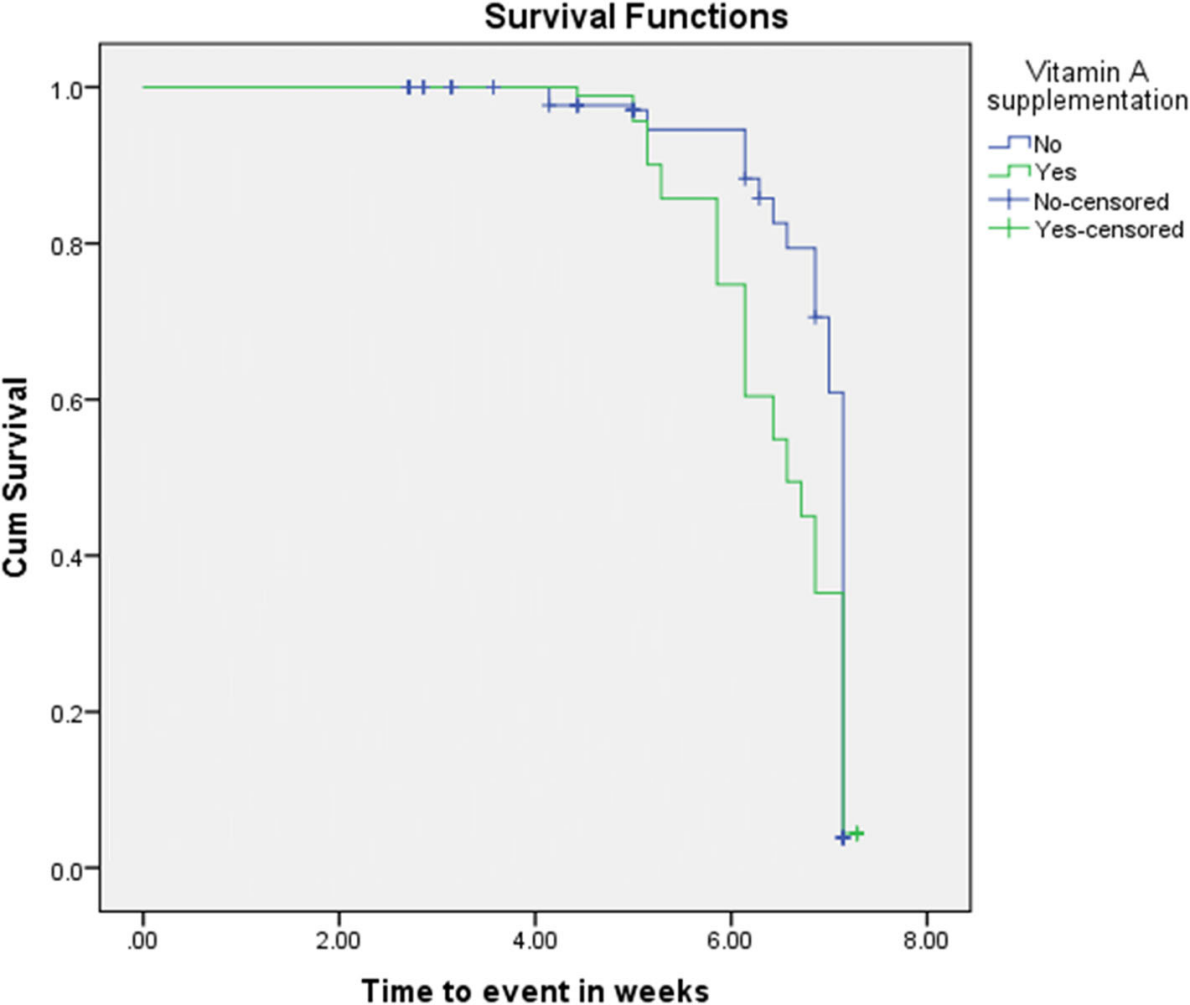
Kaplan-Meier survival curves by child’s Vitamin A supplementation in Afar Regional State, Ethiopia, 2017

The KM survival curve of vitamin A supplementation participants in relation to time to event shows that children who received vitamin A supplementation had better treatment outcomes of OTP (cure rate of 97.9.0%, median length of stay of 47 days, and mean weight gain of 11.04 g/kg/day) as compared with children who were not supplemented (cure rate of 68.3%, median length of stay of 50 days, and mean weight gain of 9.95 g/kg/day) (Fig. 2).

## Discussion

This study showed that 83.2% (95% CI (79, 88) cases were cured. This is higher than sphere acceptable range [1]. This is similar with the UNICEF report of 80.7% recovery rate from pastoral setup in Kenya [14]. However, it is higher than the rates reported from Enderta District (76.8%) [20], 67.7% from Kamba District [21], 64.9% at Wolaita Zone [22], and 61.78% in Tigray region of Ethiopia [23]. This study also revealed that 6.3% and 2.8% of children defaulted and died, respectively. This is lower than the sphere standard acceptable ranges: < 10% death and < 15% defaulters [1] (Table 4). This shows that outpatient therapeutic feeding program is an effective intervention in the treatment of severe acute malnutrition even in poor environmental settings. Hence, increasing the capacity of health institutions can improve the effectiveness and impact of the interventions.

**Table 4:** Performance indicators of OTP and sphere standard references in Afar Regional State, Ethiopia, 2017

Performance indicators	Frequencies of indicators	Sphere standards	
		Acceptable (%)	Alarming (%)
Recovery rate	83.2	> 75	< 50
Death rate	2.8	< 10	> 15
Default rate	6.3	< 15	< 25
Average weight gain	10.5 g/kg/days	≥ 8 g/kg/days	< 8 g/kg/days
Length of stay	6.3 weeks	< 4 weeks	> 6 weeks

The recovery rate of children whose mothers travel less than 2 h to the health institutions was about three times higher than children whose mothers travel 2 h and above. This is in line with the findings at Enderta district of Northern Ethiopia where the rate of recovery from OTP among children whose mothers travel below 2 h to the health facility was higher than that of children whose mothers travel 2 h and above [20]. The finding of this study can be possibly explained by the lack of consistent attendance of children residing in longer distance from OTP sites. Despite the default rate being acceptable, children reside in the longer distance were less likely to come OTP site regularly per week than living in near the site. This implies that providing either the intervention (OTP sites) closer to the community or transportation will improve the recovery rate of children.

Compared with children who received vitamin A supplementation, children who lack supplementation were 61% less likely to be cured. This is relatively similar with findings at Sekota Hospital where cure rate among children who were not supplemented with vitamin A was 53% less than supplemented children [24]. Vitamin A is required for the integrity of epithelial cells in the body as well as in the maintenance of immune function. Therefore, vitamin A is essential to combat infections and risk of illness and death from childhood infections.

The rate of recovery from OTP among children who received antibiotics was about 1.4 times higher compared with children who did not receive antibiotics. Similar findings were reported at Wolaita Zone [22], Tigray region [23], and Sekota hospital [24]. Small bowel bacterial overgrowth occurs in all children with SAM. These enteric bacteria frequently are the source of systemic infection by translocation across the bowel wall. They also cause mal-absorption of nutrients, failure to eliminate substances excreted in the bile, fatty liver, and intestinal damage, and can cause chronic diarrhea [7]. Therefore, in this study better recovery rate of children provided with antibiotics is explained by the supportive effect of antibiotics in the treatment of small bowel bacterial overgrowth.

Since this study was a prospective cohort study, temporality cause and effect relationship was possible to establish for the study factors. The treatment outcome indicators have been appropriately described, as a maximum effort was made to know the right treatment outcome of children who were lost to follow-up through a home visit.

## Conclusion

This study showed that nearly eight children in every ten had recovered from severe acute malnutrition. Children whose mothers travel less than 2 h and who received vitamin A supplementation and antibiotics had better recovery rate. Therefore, considering the distance of health facility from children’s residence, improving vitamin A supplementation and antibiotics are vital to promote the recovery rate of children from severe acute malnutrition. Further research is also required to identify and address barriers to the provision of antibiotics and vitamin A supplementation.

## Data Availability

The findings were declared from the available data source. All possible required information is included in the manuscript. Moreover, raw data is available on the hand of the corresponding author. All coauthors gave full responsibility for the corresponding author to share and/or discuss with editors and reviewers.
